# The role of cell-free hemoglobin and haptoglobin in acute kidney injury in critically ill adults with ARDS and therapy with VV ECMO

**DOI:** 10.1186/s13054-022-03894-5

**Published:** 2022-02-22

**Authors:** Jan A. Graw, Philip Hildebrandt, Alexander Krannich, Felix Balzer, Claudia Spies, Roland C. Francis, Wolfgang M. Kuebler, Steffen Weber-Carstens, Mario Menk, Oliver Hunsicker

**Affiliations:** 1 Department of Anesthesiology and Operative Intensive Care Medicine CCM / CVK, Charité – Universitätsmedizin Berlin, Corporate Member of Freie Universität Berlin, Humboldt-Universität Zu Berlin, and Berlin Institute of Health, Augustenburger Platz 1, 13353 Berlin, Germany; 2 ARDS/ECMO Centrum Charité, Charité - Universitätsmedizin Berlin, Berlin, Germany; 3 Berlin Institute of Health (BIH), Berlin, Germany; 4 Clinical Trial Office, Charité - Universitätsmedizin Berlin, Berlin, Germany; 5 Institute of Medical Informatics, Charité - Universitätsmedizin Berlin, Berlin, Germany; 6 Institute of Physiology, Charité - Universitätsmedizin Berlin, Berlin, Germany

**Keywords:** Cell-free hemoglobin, Haptoglobin, Acute kidney injury, ARDS

## Abstract

**Background:**

Increased plasma concentrations of circulating cell-free hemoglobin (CFH) are supposed to contribute to the multifactorial etiology of acute kidney injury (AKI) in critically ill patients while the CFH-scavenger haptoglobin might play a protective role. We evaluated the association of CFH and haptoglobin with AKI in patients with an acute respiratory distress syndrome (ARDS) requiring therapy with VV ECMO.

**Methods:**

Patients with CFH and haptoglobin measurements before initiation of ECMO therapy were identified from a cohort of 1044 ARDS patients and grouped into three CFH concentration groups using a risk stratification. The primary objective was to assess the association of CFH and haptoglobin with KDIGO stage 3 AKI. Further objectives included the identification of a target haptoglobin concentration to protect from CFH-associated AKI.

**Measurements and main results:**

Two hundred seventy-three patients fulfilled the inclusion criteria. Of those, 154 patients (56.4%) had AKI at ECMO initiation. The incidence of AKI increased stepwise with increasing concentrations of CFH reaching a plateau at 15 mg/dl. Compared to patients with low [< 5 mg/dl] CFH concentrations, patients with moderate [5–14 mg/dl] and high [≥ 15 mg/dl] CFH concentrations had a three- and five-fold increased risk for AKI (adjusted odds ratio [OR] moderate vs. low, 2.69 [95% CI, 1.25–5.95], *P* = 0.012; and OR high vs. low, 5.47 [2.00–15.9], *P* = 0.001). Among patients with increased CFH concentrations, haptoglobin plasma levels were lower in patients with AKI compared to patients without AKI. A haptoglobin concentration greater than 2.7 g/l in the moderate and 2.4 g/l in the high CFH group was identified as clinical cutoff value to protect from CFH-associated AKI (sensitivity 89.5% [95% CI, 83–96] and 90.2% [80–97], respectively).

**Conclusions:**

In critically ill patients with ARDS requiring therapy with VV ECMO, an increased plasma concentration of CFH was identified as independent risk factor for AKI. Among patients with increased CFH concentrations, higher plasma haptoglobin concentrations might protect from CFH-associated AKI and should be subject of future research.

**Supplementary Information:**

The online version contains supplementary material available at 10.1186/s13054-022-03894-5.

## Introduction

Elevated plasma levels of circulating cell-free hemoglobin (CFH) can be found in patients with sepsis or after cardiac surgery and seem to be associated with a worse short-term outcome [1–5]. Likewise, observational studies in cardiac surgery patients suggest an association between increased plasma concentrations of CFH and acute kidney injury (AKI) [3, 6].

The mechanisms of toxicity caused by CFH include nitric oxide scavenging with concomitant vasoconstriction, platelet aggregation, inflammation, lipid peroxidation, mitochondrial damage, increased oxidative stress, and stimulation of pro-inflammatory receptors [7–10]. While renal damage is commonly attributed to general or regional renal hypoperfusion, increased levels of circulating CFH lead to glomerular filtration of CFH which may further aggravate renal damage by subsequent tubular injury [6]. As a result, increased plasma concentrations of CFH might contribute to the multifactorial etiology of AKI in critically ill patients. However, to date, there is limited clinical data evaluating CFH as a risk factor for AKI in critical ill patients.

The endogenous CFH-scavenger haptoglobin removes CFH from plasma by forming high-molecular-weight haptoglobin-hemoglobin complexes that bind to the CD 163 receptor on hepatic and splenic macrophages [11]. Experimental data suggest that by binding CFH-dimers into a haptoglobin-hemoglobin complex, haptoglobin prevents glomerular filtration of CFH and protects from subsequent kidney injury [12, 13]. The therapeutic use of plasma-purified haptoglobin in humans is limited to case reports where haptoglobin is used to reduce renal exposure to CFH in cardiac surgery [14, 15]. Currently, no specific disease state with measurable efficacy endpoints exists to study administration of exogenous haptoglobin as a therapeutic option with regard to safety and efficacy [16].

The objectives of this study were therefore to investigate if CFH is a risk factor for AKI in a large cohort of patients with ARDS requiring therapy with VV ECMO and to probe for a potentially protective role of haptoglobin in patients with increased CFH concentrations. Furthermore, we aimed to provide cut-off values for a haptoglobin plasma concentration to protect from CFH-associated AKI that might serve as a target concentration for therapy with exogenous haptoglobin.

## Methods

### Study design and setting

This is a retrospective cohort study of ARDS patients who received treatment with VV ECMO at the tertiary ARDS referral center of the Department of Anesthesiology and Intensive Care Medicine, Charité–Universitätsmedizin Berlin, Campus Virchow-Klinikum, Berlin between January 2007 and December 2018. Standards for monitoring of ECMO patients at the Charité ARDS/ECMO center include daily measurements of CFH and haptoglobin plasma concentrations. This study investigated the association of CFH and haptoglobin with incidence of AKI at ECMO initiation. According to the baseline CFH plasma concentrations before ECMO initiation, patients were grouped into a low, moderate, and high CFH group using a risk stratification. After comparing patients’ characteristics between these groups, the CFH concentration as a risk factor for AKI at ECMO initiation was evaluated. Thereafter, the role of haptoglobin in patients with increased CFH concentrations was assessed, and clinical cut-off values to protect from AKI were identified. The Medical Ethics Committee of the Charité *–* Universitätsmedizin Berlin approved the study (No. EA2/019/19).

### Participants

All adult patients fulfilling the criteria of the Berlin Definition for ARDS and requiring VV ECMO at the tertiary ARDS referral center of the Department of Anesthesiology and Intensive Care Medicine, Charité *–* Universitätsmedizin Berlin, Campus Virchow-Klinikum, Berlin between January 2007 and December 2018 were enrolled into the study [17]. Patients were excluded if they had no baseline measurements of CFH before ECMO initiation.

### Laboratory measurements and grouping

Plasma obtained from EDTA blood for standard laboratory tests and centrifuged within two hours (15 min, 2500 g) was assessed for CFH and haptoglobin, respectively. Plasma levels of CFH were measured by photometry, and plasma levels of haptoglobin were measured by turbidimetry, both with a cobas 8000 modular analyzer series system (Roche Diagnostics, Basel). According to the baseline CFH plasma concentrations before ECMO initiation, patients were grouped into a low, moderate, and high CFH group using a risk stratification based on a gray zone approach [18]. This approach allows for a clinically meaningful grouping by providing a CFH group that will not experience the event (occurrence of AKI) with near certainty (the low CFH group), a CFH group that will experience the event (occurrence of AKI) with near certainty (the high CFH group), and a CFH group that corresponds to a range of CFH concentrations for which formal conclusions could initially not be obtained, the so-called gray zone (the moderate CFH group). For this purpose, two cut-offs are calculated that are used to separate the low and moderate as well as the moderate and high CFH concentration group. A high sensitivity of 90% was chosen to provide the cut-off that allows exclusion of the diagnosis with near certainty (no AKI), while a high specificity of 90% was chosen to provide the cut-off that includes the diagnosis with near certainty (occurrence of AKI). The high sensitivity cut-off was used to separate the low and moderate CFH concentration group, while the high specificity cut-off was used to separate the moderate and high CFH concentration group.

### Objectives, bias handling, and data sources

The primary objective was to investigate the role of the CFH concentration as a risk factor for KDIGO stage 3 AKI at ECMO initiation. Kidney Disease: Improving Global Outcomes (KDIGO) stage 3 AKI was defined as increase in serum creatinine level by ≥ 4.0 mg/dl and/or urine output < 0.3 ml/kg/h for > 24 h or anuria for > 12 h and/or acute initiation of renal replacement therapy (RRT). To address a potential selection bias, relevant baseline characteristics and other known risk factors for AKI were considered. Secondary objectives included evaluation of the role of the CFH-scavenger haptoglobin in patients with increased CFH concentrations and identification of clinical cut-off values for haptoglobin to protect from AKI. All data required for this study were extracted from the two electronic patient data management systems in use at the hospital (COPRA, Sabachswalden, Germany; and SAP, Walldorf, Germany). Further details are provided in the Supplemental methods (Additional file 1).

### Statistical analyses

Differences of continuous data were tested using the exact Mann–Whitney U test and the Kruskal–Wallis test, and frequencies were tested using Fisher’s exact test.

To evaluate the association of the CFH concentration with AKI at ECMO initiation, a multivariable logistic regression was performed using a backward variable selection procedure based on the Akaike information criterion (AIC). Baseline characteristics that were different in univariable analysis, and known risk factors were included, as appropriate. The area under the curve was used to measure internal validation. The net reclassification improvement (NRI) was calculated to assess if the CFH concentration provides additional predictive value [19, 20]. The NRI determination was based on the risk stratification. The NRI determines the improvement regarding correct upward and downward reclassification of predicted probabilities when a new determinant is added to a pre-existing risk prediction model.

To identify a target haptoglobin concentration to protect from CFH-associated AKI, haptoglobin was compared between patients with and without AKI in the moderate and high CFH group. Thereafter, sensitivity and specificity analyses were performed to identify clinical cut-off values of interest. A high specificity cut-off of 90% was chosen as clinical cut-off where CFH-associated AKI occurs with near certainty while a high sensitivity cut-off of 90% was chosen as clinical cut-off to protect from CFH-associated AKI. Due to the exploratory study type, all analyses were considered to be non-confirmatory. A two-tailed *P* value < 0.05 was considered statistically significant. Statistical analyses were performed with the use of R software, version 3.6.1 (R Project for Statistical Computing, Vienna, Austria).

## Results

**Fig. 1:**
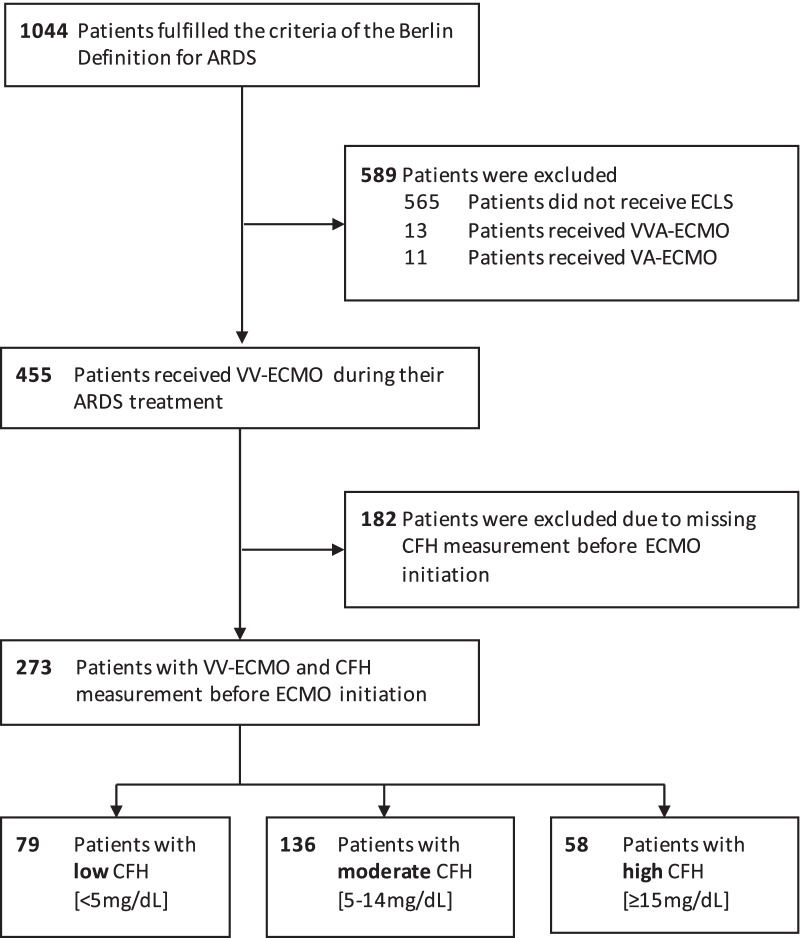
Study flow diagram

A total of 1044 patients with ARDS were identified within the analyzed time period. Of the 455 ARDS patients that received therapy with VV ECMO, 273 patients had CFH measurements before ECMO initiation (Fig. 1). Patients had an average PaO_2_:FiO_2_ ratio of 70 mmHg (IQR 54–89), and the distribution of ARDS etiology was consistent with previously published cohorts (Table 1). Simultaneous measurements of haptoglobin were obtained in 257 patients (94.1%). The distribution of CFH and haptoglobin concentration in the population is provided in Figure S1 and S2 (Additional file 1).

**Table 1:** Patients characteristics

Characteristic	Low CFH [< 5 mg/dl] (*N* = 79)	Moderate CFH [5–14 mg/dl] (*N* = 136)	High CFH [≥ 15 mg/dl] (*N* = 58)	*P* value*
CFH concentration	3.00 [2.00, 3.60]	8.00 [6.00, 10.25]	23.00 [17.25, 42.25]	< 0.001
Age (years)	48.00 [37.50, 64.00]	53.00 [40.00, 64.00]	46.00 [28.50, 55.75]	0.030
Male sex, *n* (%)	58 (73.4)	87 (64.0)	38 (65.5)	0.351
Body mass index (kg/cm)	24.84 [21.60, 27.78]	26.12 [22.17, 29.93]	24.49 [21.57, 30.96]	0.454
Charlson comorbidity index	2.00 [0.50, 5.00]	3.00 [1.00, 4.00]	2.00 [0.00, 4.00]	0.359
Chronic kidney disease, *n* (%)	6 (7.6)	13 (9.6)	6 (10.3)	0.837
Immunocompromised, *n* (%)	19 (24.1)	38 (27.9)	15 (25.9)	0.819
SOFA at ARDS onset	11.00 [8.00, 14.00]	12.00 [9.75, 15.00]	14.50 [11.25, 16.75]	< 0.001
SAPS II at ARDS onset	49.00 [35.50, 63.50]	58.00 [42.00, 70.25]	59.50 [47.00, 77.00]	0.005
Chronic lung disease, *n* (%)	32 (40.5)	51 (37.5)	16 (27.6)	0.273
Pulmonary origin, *n* (%)	74 (93.7)	120 (88.2)	44 (75.9)	0.008
ARDS severity, *n* (%)				
Severe	76 (96.2)	130 (95.6)	56 (96.6)	0.945
ARDS etiology, *n* (%)				0.387
Pneumonia	54 (70.1)	92 (70.2)	32 (55.2)	
Aspiration pneumonitis	5 (6.5)	13 (9.9)	8 (13.8)	
Trauma and burns	1 (1.3)	5 (3.8)	2 (3.4)	
Other acute respiratory diagnoses	6 (7.8)	11 (8.4)	8 (13.8)	
Nonrespiratory and chronic respiratory diagnoses	11 (14.3)	10 (7.6)	8 (13.8)	
ECMO initiation, *n* (%)				0.705
Mobile ECMO retrieval team	20 (25.3)	35 (25.7)	18 (31.0)	
After admission	59 (74.7)	101 (74.3)	40 (69.0)	
ECMO initiation (ICU day)	0.00 [0.00, 0.00]	0.00 [0.00, 0.00]	0.00 [0.00, 0.00]	0.564
Ventilation parameters at ECMO initiation				
Mechanical ventilation (days)	3.00 [1.00, 7.50]	2.00 [1.00, 5.50]	1.50 [1.00, 4.00]	0.312
PaO2:FiO_2_ (mmHg)	76.00 [58.66, 89.30]	68.49 [56.12, 90.00]	60.90 [50.76, 85.50]	0.267
PaO_2_ (mmHg)	72.50 [56.22, 85.72]	66.40 [52.10, 86.55]	57.30 [50.50, 85.50]	0.440
PaCO_2_ (mmHg)	69.00 [56.60, 88.05]	62.45 [50.35, 78.40]	71.50 [57.55, 82.40]	0.153
pH	7.25 [7.18, 7.35]	7.25 [7.17, 7.36]	7.20 [7.11, 7.27]	0.095
PIP (cmH_2_O)	36.20 [31.63, 40.25]	36.86 [32.67, 42.00]	40.50 [36.00, 47.00]	0.001
Pplateau (cmH_2_O)	33.19 [29.00, 36.02]	33.44 [30.00, 38.00]	38.00 [31.00, 41.00]	0.011
PEEP (cm H_2_O)	18.20 [15.30, 20.00]	18.20 [16.00, 20.40]	20.40 [17.00, 22.00]	0.013
Driving pressure (cmH_2_O)	15.60 [11.00, 17.07]	14.00 [11.00, 19.40]	17.00 [12.85, 20.60]	0.179
Tidal volume (ml/kg PBW)	5.32 [3.55, 7.80]	5.44 [3.88, 7.41]	5.27 [3.86, 6.96]	0.836
Respiratory rate (breaths/min)	25.00 [20.00, 34.34]	24.25 [20.00, 30.05]	25.00 [19.74, 31.79]	0.742
Compliance (ml/cm H_2_O)	24.61 [15.25, 32.00]	23.80 [13.06, 33.78]	17.09 [9.73, 22.74]	< 0.001
Further rescue therapy, *n* (%)				
Inhaled nitric oxide	54 (68.4)	95 (69.9)	41 (70.7)	0.954
Prone positioning	60 (75.9)	100 (73.5)	44 (75.9)	0.902
Organ failure at ECMO initiation, *n* (%)				
Coagulation	13 (16.5)	47 (34.6)	31 (53.4)	< 0.001
Liver	7 (8.9)	18 (13.2)	12 (20.7)	0.134
Cardiovascular	76 (100.0)	123 (100.0)	55 (100.0)	NA
CNS	42 (53.2)	71 (52.2)	27 (46.6)	0.712
Renal (KDIGO 3)	29 (36.7)	81 (59.6)	44 (75.9)	< 0.001
Septic shock, *n* (%)	34 (43.0)	79 (58.1)	39 (67.2)	0.014
RRT, *n* (%)	28 (35.4)	77 (56.6)	44 (75.9)	< 0.001
28-day mortality, *n* (%)	18 (22.8)	53 (39.0)	32 (55.2)	0.001

Data are expressed as median (25%-75% quartiles) or frequencies (%), as appropriate

CFH, cell-free hemoglobin; SOFA, Sequential Organ Failure Assessment; SAPS, Simplified Acute Physiology Score; ECMO, extracorporeal membrane oxygenation; PIP, peak inspiratory pressure; Pplateau, plateau pressure; PEEP, positive end-expiratory pressure; RRT, renal replacement therapy

*P value was calculated comparing low, moderate, and high CFH groups using the Kruskal–Wallis test and the Fisher’s exact test, as appropriate

**Fig. 2:**
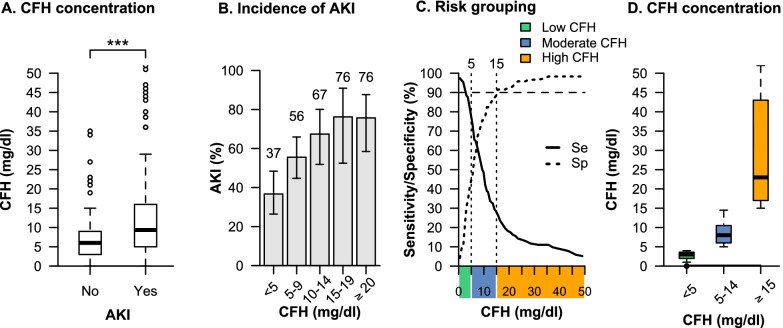
Association of CFH concentrations with AKI and grouping. Comparison of CFH concentrations between patients with and without AKI (**A**). Incidence of AKI with 95% confidence intervals across different CFH concentrations (**B**). Risk grouping according to the sensitivity and specificity analysis of CFH concentration for occurrence of AKI (**C**). Cutoffs with 90% sensitivity and 90% specificity are used to group patients into the three CFH concentrations groups. Distribution of the CFH concentration within each group (**D**). ***P < 0.001. Se, Sensitivity; Sp, Specificity

Overall, 154 patients (56.4% [95% CI, 50.2–62.3]) had KDIGO stage 3 AKI before ECMO initiation. Details on the components of KDIGO stage 3 AKI and indication for RRT are shown in Figure S3, characteristics of patients with and without AKI are shown in Table S1 (Additional file 1). Patients with AKI at ECMO initiation had higher CFH concentrations compared to patients without AKI (9.4 mg/dl [IQR 5–16] vs. 6.0 mg/dl [IQR 3–9], *P* < 0.001) (Fig. 2A). The incidence of AKI increased stepwise with increasing concentrations of CFH reaching a plateau at 15 mg/dl (Fig. 2B). According to risk stratification based on the gray zone approach, patients were grouped into a low [< 5 mg/dl], moderate [5–14 mg/dl], and high [≥ 15 mg/dl] CFH concentration group (Fig. 2C). Seventy-nine patients (28.9%) had low, 136 patients (49.8%) had moderate, and 58 patients (21.2%) had high CFH concentrations. The corresponding median CFH concentrations in the low, moderate, and high CFH concentration group were 3 mg/dl (IQR 2–4]), 8 mg/dl (IQR 6–11), and 23 mg/dl (IQR 17–43), respectively (Fig. 2D).

Characteristics of the patients after CFH grouping are shown in Table 1. Patients with higher CFH concentrations had higher SOFA and SAPS II scores and a higher prevalence of septic shock at ECMO initiation. There were no differences in demographic data, comorbidities, ARDS severity, ARDS etiology, time of ECMO initiation, and the use of rescue therapies such as therapy with inhaled nitric oxide or prone positioning.

**Fig. 3:**
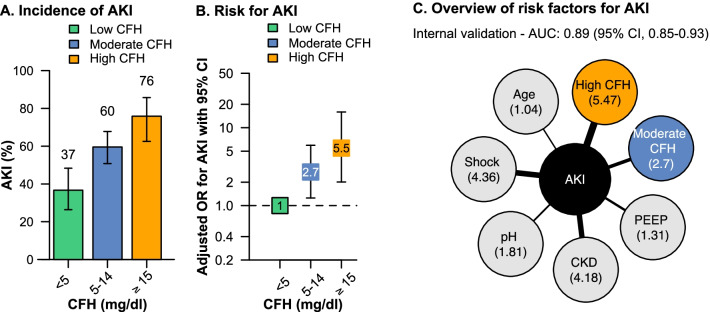
The CFH concentration as a risk factor for AKI. Incidence of AKI with 95% confidence intervals in the three CFH concentrations groups (**A**). Vertically aligned forest plot presenting the adjusted chances (adjusted odds ratios with 95% confidence intervals) for AKI at ECMO initiation in the low, moderate, and high CFH group (**B**). The low CFH group was set as the reference. Overview and adjusted odds ratios (given in brackets without 95% confidence intervals) of all independent risk factors for AKI (**C**) obtained from multivariable logistic regression model

Incidences of AKI were 36.7% (95% CI, 26.3–48.3) in the low, 59.5% (95% CI 50.7–67.7) in the moderate, and 75.8% (95% CI, 62.5–85.7) in the high CFH concentration group (*P* < 0.001) (Fig. 3A). Compared to patients with low CFH concentrations, patients with moderate concentrations had a two and a half-fold increased risk of AKI (odds ratio [OR] moderate vs. low, 2.53 [95% CI, 1.44–4.53], *P* < 0.001). Patients with high CFH concentrations had a five-fold increased risk of AKI compared to patients with low CFH concentrations (OR high vs. low, 5.41 [95% CI, 2.59–11.8], *P* < 0.001) (Table 2). These findings were confirmed in a multivariable model adjusting for other independent risk factors such as age, chronic kidney disease, pH, PEEP, and septic shock. Internal validation of the model demonstrated an excellent discrimination for AKI (c = 0.89 [95% CI, 0.85–0.93]) (Table 2, Fig. 3B, C). Adding the three-stage risk classification of CFH to the clinical risk prediction model for KDIGO stage 3 AKI at ECMO initiation (based on age, chronic kidney disease, pH, PEEP, and septic shock) resulted in a substantial reclassification improvement in patients developing AKI (NRI for events 57%, *P* < 0.001).

**Table 2:** Assessment of risk factors for KDIGO stage 3 AKI at ECMO initiation in univariable and multivariable analysis

Model and variables	Univariable model Odds ratio (95% CI)	*P* value	Multivariable model* Odds ratio (95% CI)	*P* value
*Model 1*				
CFH				
Low CFH	Reference		Reference	
Moderate CFH	2.53 (1.44–4.53)	< 0.001	2.69 (1.25–5.95)	0.012
High CFH	5.41 (2.59–11.8)	< 0.001	5.47 (2.00–15.9)	0.001
Age	1.02 (1.01–1.04)	0.008	1.03 (1.01–1.06)	0.001
Chronic lung disease	0.35 (0.21–0.59)	< 0.001	–	
Chronic kidney disease	3.20 (1.71–8.05)	0.001	4.18 (1.75–15.6)	0.007
Pulmonary origin	0.40 (0.17–0.86)	0.03	–	
ARDS severity	6.21 (1.59–41.3)	0.02	–	
pH at ECMO initiation	0.94 (0.91–0.96)	< 0.001	1.80 (1.30–2.56)	< 0.001
PIP at ECMO initiation (per cmH2O)	1.07 (1.04–1.11)	< 0.001	–	
Pplateau at ECMO initiation (cmH2O)	1.10 (1.06–1.15)	< 0.001	–	
PEEP at ECMO initiation (per cmH2O)	1.29 (1.20–1.41)	< 0.001	1.31 (1.19–1.46)	< 0.001
Septic shock	5.39 (3.21–9.20)	< 0.001	4.35 (2.18–8.97)	< 0.001
*Model 2*				
CFH and Haptoglobin				
Low CFH	Reference		Reference	
Moderate CFH, Haptoglobin ≥ 2.7 g/l	1.72 (0.57–5.16)	0.32	1.17 (0.24–5.44)	0.11
Moderate CFH, Haptoglobin < 2.7 g/l	2.60 (1.44–4.75)	0.001	3.08 (1.37–7.12)	0.007
High CFH, Haptoglobin ≥ 2.4 g/l	3.44 (0.63–25.9)	0.17	2.89 (0.44–24.9)	0.27
High CFH, Haptoglobin < 2.4 g/l	7.08 (3.10–17.5)	< 0.001	9.22 (2.80 -34.4)	< 0.001
Age	See Model 1		1.04 (1.02–1.06)	0.001
Chronic lung disease	See Model 1		-	
Chronic kidney disease	See Model 1		4.74 (1.84–18.5)	0.005
Pulmonary origin	See Model 1		-	
ARDS severity	See Model 1		-	
pH at ECMO initiation	See Model 1		1.77 (1.26–2.55)	0.001
PIP at ECMO initiation (per cmH2O)	See Model 1		–	
Pplateau at ECMO initiation (cmH2O)	See Model 1		–	
PEEP at ECMO initiation (per cmH2O)	See Model 1		1.34 (1.21–1.52)	< 0.001
Septic shock	See Model 1		3.97 (1.94–8.39)	< 0.001

CFH, cell-free hemoglobin; ECMO, extracorporeal membrane oxygenation; PIP, peak inspiratory pressure; Pplateau, plateau pressure; PEEP, positive end-expiratory pressure

^*^Multivariable logistic regression analyses using a backward variable selection procedure based on the Akaike information criterion (AIC). According to AIC analyses, the following variables were removed from the models: Chronic lung disease, Pulmonary origin, ARDS severity, PIP at ECMO initiation, and Pplateau at ECMO initiation. Internal validation demonstrated an excellent discrimination for model 1 c = 0.89 [95% CI, 0.85–0.93] and model 2 c = 0.90 [95% CI, 0.86–0.93]

**Fig. 4:**
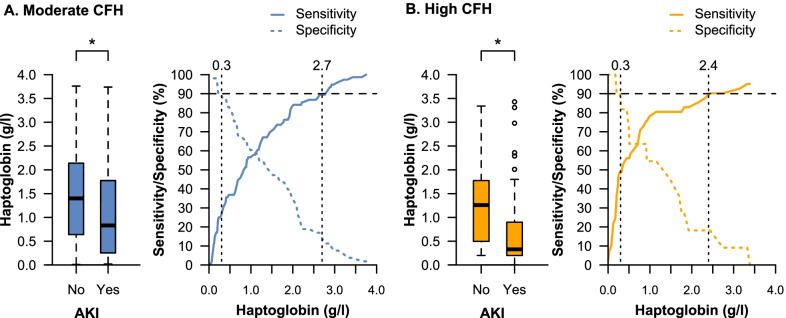
The role of haptoglobin in patients with increased CFH concentrations. Haptoglobin concentrations of patients with and without AKI among patients with moderate (**A**) and high (**B**) CFH concentrations. Corresponding sensitivity and specificity analysis of haptoglobin concentration for occurrence of AKI within the moderate (**A**) and high (**B**) CFH concentration group are indicated. Clinical cutoffs with 90% sensitivity and 90% specificity are indicated. In patients with moderate CFH, *n* = 16 (12.4%) had haptoglobin concentrations > 2.7 g/l while *n* = 27 (20.9%) had haptoglobin concentrations < 0.3 g/l. In patients with high CFH, *n* = 6 (11.5%) had haptoglobin concentrations > 2.4 g/l while *n* = 21 (40.3%) had haptoglobin concentrations < 0.3 g/l. *P < 0.05

Among patients with moderate and high CFH concentrations, haptoglobin plasma concentrations were significantly lower in patients with AKI at ECMO initiation compared to patients without AKI (Fig. 4). Patients without AKI in the moderate CFH group had similar haptoglobin concentrations compared to patients without AKI in the high CFH group (1.4 g/l [IQR 0.6–2.1] vs. 1.3 g/l [IQR 0.5–1.8], *P* = 0.53). A haptoglobin concentration greater than 2.7 g/l in the moderate CFH group and greater than 2.4 g/l in the high CFH group was identified as a clinical cutoff value to protect from CFH-associated AKI with near certainty (sensitivity 89.5% [95% CI, 83–96] and 90.2% [95% CI, 80–97], respectively). Furthermore, the sensitivity curves in the moderate and high CFH group indicate that even with lower CFH concentrations such as 2 g/l, the risk for AKI is still low (sensitivity > 80%). In contrast, patients with a haptoglobin concentration lower than 0.3 g/l developed AKI with near certainty in both groups (specificity 89% [95% CI, 79–96], and 91% [95% CI, 73–99], respectively).

**Fig. 5:**
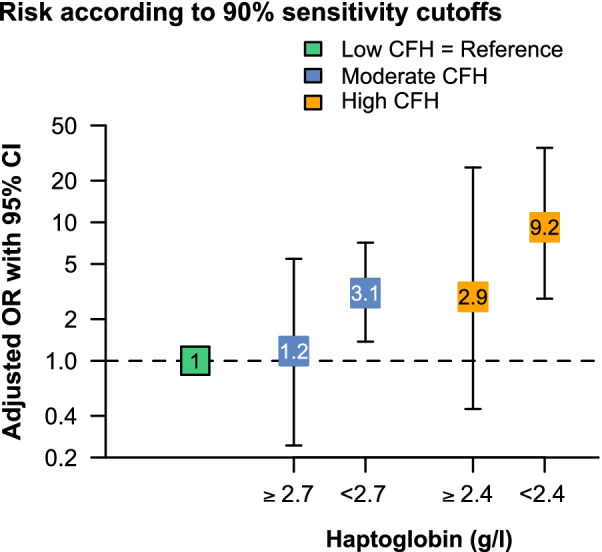
The CFH concentration as a risk factor for AKI considering different corresponding haptoglobin concentrations. Vertically aligned forest plot presenting the adjusted chances (adjusted odds ratios with 95% confidence intervals) for AKI at ECMO initiation in the low, moderate, and high CFH group. The moderate and high CFH groups were separated according to the particular 90% sensitivity cutoffs in each group. The low CFH group was set as the reference

Using the identified clinical cutoff values to protect from CFH-associated AKI with near certainty indicated that patients with moderate CFH concentrations and haptoglobin concentrations greater than 2.7 g/l had a similar risk for AKI as patients with low CFH concentrations (OR moderate vs. low, 1.17 [95% CI, 0.24–5.44], *P* = 0.83) (Fig. 5). In patients with high CFH concentrations, haptoglobin concentrations greater than 2.4 g/l attenuated the risk for AKI when compared to patients with low CFH concentrations (OR high vs. low, 2.89 [95% CI, 0.44–24.9], *P* = 0.27).

## Discussion

In patients with ARDS requiring therapy with VV ECMO, the incidence of KDIGO stage 3 AKI was independently associated with elevated plasma concentrations of CFH and increased linearly as a function of CFH plasma concentration reaching a plateau at 15 mg/dl. Among patients with high and moderate CFH concentrations, we could determine clinical cutoff values of 2.4 g/l and 2.7 g/l, respectively, for a plasma haptoglobin concentration to protect from CFH-associated AKI.

The importance of protective mechanisms against the adverse effects of intravascular hemolysis in organisms with a blood circulation is highlighted by the evolutionary early appearance and conservation of haptoglobin [21]. Clinical data have shown an association between increased plasma levels of CFH, decreased concentrations of haptoglobin and mortality for patients with sepsis [2, 22, 23]. The cause of increased erythrocyte fragility in sepsis is so far not understood and appears to be of multifactorial origin [2, 22, 23]. While pathogens or their toxins can affect the integrity of the red cell membrane, CFH may also originate from direct mechanical injury of red cells in the vasculature. In addition, extracorporeal therapeutic measures such as ECMO can cause mechanical hemolysis [24]. To control for the impact of ECMO therapy on hemolysis in patients with ARDS, only measurements of CFH and haptoglobin at the time of ECMO initiation were included in the present analysis.

The injurious potential of intravascular CFH manifests most prominently in the kidney [3, 6, 25]. In cardiac surgery patients, increased plasma concentrations of CFH after prolonged cardiopulmonary bypass are associated with AKI [3]. In this population of ARDS patients, the occurrence of AKI at ECMO initiation increased in a dose dependent manner as a function of higher CFH concentrations. However, in patients with plasma haptoglobin levels in the normal range, no association between CFH and AKI at ECMO initiation was detected while patients with low haptoglobin concentrations had a dose-dependent higher risk for CFH-associated AKI. While based on association, our data might suggest a protective effect of haptoglobin for kidney function in disease conditions associated with elevated plasma CFH. The hypothesis that AKI may be prevented by treatment with exogenous haptoglobin is underlined by several preclinical studies demonstrating that supplementing haptoglobin in conditions of hemolysis could protect from CFH-associated renal injury [12, 26]. With CFH, haptoglobin forms one of the strongest protein-complexes in plasma [27]. Specifically, administration of exogenous haptoglobin can prevent renal filtration of CFH and thus, subsequent tubular injury, and preserves vascular function during hemolysis [12, 26, 28–30].

Despite the lack of large prospective trials, treatment with haptoglobin is already in clinical use in Japan to prevent hemoglobinuria and potentially AKI in severe hemolysis after CPB [14, 15]. However, dosing of haptoglobin is guided by an effect-approach with increasing haptoglobin doses until hemoglobinuria suspends and not by haptoglobin plasma target concentrations [16]. For approval of haptoglobin supplementation as a therapeutic intervention, a safety and efficacy trial in a specific disease state with a measureable efficacy end point would be required [16]. In this study, we identified AKI in ARDS as such a potential end point and disease state combination for potential future prospective trials. Therefore, the haptoglobin plasma concentration with the associated AKI-protective clinical cutoff values might not only be studied as a future additional marker for disease severity and risk for AKI in patients with ARDS but also as a potential target for treatment with exogenous haptoglobin in future prospective clinical trials.

This study has several limitations. Based on the availability of data on CFH and haptoglobin plasma concentrations, only patients admitted with severe ARDS and ECMO treatment were included. Standards for monitoring ECMO patients at our center include regular full blood counts, coagulation measurements and daily measurements of CFH and haptoglobin plasma concentrations. However, as daily CFH and haptoglobin measurements are performed to monitor functionality of the ECMO system, not all patients had CFH and haptoglobin plasma concentrations measured before ECMO initiation. Interestingly, ARDS etiology was not associated with presence of hemolysis at ECMO initiation. The potential selection of very sick ARDS patients by our inclusion criteria is underscored by the high rate of 56.4% of patients with AKI at ECMO initiation. Due to the retrospective design of this study, no causative conclusions can be drawn from the current observations. Although multiple adjustments for important prognostic determinants were made, and high quality criteria such as sensitivity and specificity cut-off greater than 90% were used, increased CFH and decreased haptoglobin plasma concentrations might only represent markers of disease severity rather than pathophysiological contributors to the disease and its complications. Prospective data are needed to shed light on the downstream effects of increased CFH plasma concentrations.

## Conclusions

In critically ill patients with ARDS, VV ECMO therapy, and elevated concentrations of circulating CFH, increased plasma-levels of haptoglobin are independently associated with a lower rate of AKI. Development of rapid bedside testing tools for CFH as well as for plasma haptoglobin and the therapeutic potential of supplementation with exogenous haptoglobin should be addressed in future clinical trials in patients with severe ARDS.

## Supplementary Information

**Additional file 1: Supplemental Methods**. Data sources, **Table S1**. Characteristics between patients with and without AKI at ECMO initiation, **Figure S1**. Distribution of CFH concentration of the study population, **Figure S2**. Distribution of haptoglobin concentration of the study population, **Figure S3**. Detailed overview of KDIGO 3 AKI components and indications for RRT in patients with acute RRT at ECMO initiation.

## Data Availability

Data are available from the corresponding author on reasonable request.
