# Supplementary material for: The role of cell-free hemoglobin and haptoglobin in acute kidney injury in critically ill adults with ARDS and therapy with VV ECMO
Source: Crit Care. 2022 Feb 22;26:50. doi: 10.1186/s13054-022-03894-5 (PMC8864920; doi:10.1186/s13054-022-03894-5)
Supplement: Supplementary file 1 — Additional file 1: Supplemental Methods. Data sources, Table S1. Characteristics between patients with and without AKI at ECMO initiation, Figure S1. Distribution of CFH concentration of the study population, Figure S2. Distribution of haptoglobin concentration of the study population, Figure S3. Detailed overview of KDIGO 3 AKI components and indications for RRT in patients with acute RRT at ECMO initiation. [file 13054_2022_3894_MOESM1_ESM.docx]

**Additional file 1**

The role of Cell-Free Hemoglobin and Haptoglobin in Acute Kidney Injury in Critically Ill Adults with ARDS and therapy with VV ECMO

J. A. Graw, P. Hildebrandt, A. Krannich, C. Spies, R. C. Francis, W.M. Kuebler, S. Weber-Carstens, M. Menk, O. Hunsicker

**Table of Contents**

[**Supplemental Methods** 2](#_Toc84798062)

[Data sources 2](#_Toc84798063)

[**Supplemental Tables** 3](#_Toc84798064)

[Table S1. Characteristics between patients with and without AKI at ECMO initiation. 3](#_Toc84798065)

[**Supplemental Figures** 4](#_Toc84798066)

[Figure S1: Distribution of CFH concentration of the study population. 4](#_Toc84798067)

[Figure S2: Distribution of haptoglobin concentration of the study population. 4](#_Toc84798068)

[Figure S3: Detailed overview of KDIGO 3 AKI components and indications for RRT in patients with acute RRT at ECMO initiation. 5](#_Toc84798069)

# Supplemental Methods

**Data sources**

Data on patients’ demographics and comorbidities were extracted from the hospital data management system (SAP, Walldorf, Germany). CFH and haptoglobin measurements, laboratory measurements, data on admission scores, ARDS characteristics, ARDS treatment, rescue therapies, supportive therapies, medications, ventilation parameters, and ECMO were extracted from the electronic intensive care unit data management system in use at the hospital (COPRA 5, Sasbachwalden, Germany).

# Supplemental Tables

**Table S1. Characteristics between patients with and without AKI at ECMO initiation.**

| **Characteristic** | **No AKI** (N=119) | **KDIGO stage 3 AKI** (N=154) | P Value* |
| --- | --- | --- | --- |
| **Age (years)** | 46.00 [32.00, 58.00] | 53.50 [41.00, 64.00] | 0.004 |
| **Male sex, n (%)** | 78 (65.5) | 105 (68.2) | 0.742 |
| **Body mass index (kg/cm)** | 24.03 [20.47, 27.93] | 26.12 [22.86, 30.86] | 0.009 |
| **Charlson comorbidity index** | 2.00 [0.00, 4.50] | 3.00 [1.00, 4.00] | 0.496 |
| **Chronic kidney disease, n (%)** | 2 (1.7) | 23 (14.9) | <0.001 |
| **Immunocompromised, n (%)** | 37 (31.1) | 35 (22.7) | 0.157 |
| **Previous chemotherapy, n (%)** | 12 (10.1) | 14 (9.1) | 0.945 |
| **SOFA at ARDS onset** | 10.00 [8.00, 12.00] | 14.00 [11.00, 17.00] | <0.001 |
| **SOFA without renal items at ARDS onset** | 10.00 [8.00, 12.00] | 13.00 [10.00, 15.00] | <0.001 |
| **SAPS II at ARDS onset** | 46.00 [35.00, 61.00] | 63.50 [48.00, 78.75] | <0.001 |
| **Chronic lung disease, n (%)** | 59 (49.6) | 40 (26.0) | <0.001 |
| **Severe ARDS (Berlin definition), n (%)** | 110 (92.4) | 152 (98.7) | 0.021 |
| **ARDS etiology, n (%)** |  |  | 0.117 |
| Pneumonia | 78 (67.8) | 100 (66.2) |  |
| Aspiration pneumonitis | 6 (5.2) | 20 (13.2) |  |
| Trauma and burn | 4 (3.5) | 4 (2.6) |  |
| Other acute respiratory diagnoses | 15 (13.0) | 10 (6.6) |  |
| Nonrespiratory and chronic respiratory diagnoses | 12 (10.4) | 17 (11.3) |  |
| **ECMO initiation, n (%)** |  |  | 0.142 |
| Mobile ECMO retrieval team | 26 (21.8) | 47 (30.5) |  |
| After admission | 93 (78.2) | 107 (69.5) |  |
| **ECMO initiation (ICU day)** | 0.00 [0.00, 0.00] | 0.00 [0.00, 0.00] | 0.085 |
| **Ventilation parameters at ECMO initiation** |  |  |  |
| Mechanical ventilation (days) | 2.00 [1.00, 6.00] | 2.00 [1.00, 6.00] | 0.095 |
| PaO2:FiO2 (mmHg) | 72.90 [56.20, 104.08] | 64.80 [53.80, 83.90] | 0.047 |
| PaO2 (mmHg) | 70.50 [53.70, 90.20] | 63.05 [51.30, 80.72] | 0.103 |
| PaCO2 (mmHg) | 66.90 [52.10, 87.00] | 63.80 [52.42, 79.07] | 0.510 |
| pH | 7.29 [7.21, 7.39] | 7.19 [7.10, 7.27] | <0.001 |
| PIP (cmH2O) | 35.73 [31.81, 40.00] | 38.96 [34.20, 45.00] | <0.001 |
| Pplateau (cmH2O) | 31.00 [28.00, 36.00] | 36.00 [31.00, 40.00] | <0.001 |
| PEEP (cm H2O) | 17.00 [13.23, 19.00] | 20.00 [18.00, 22.00] | <0.001 |
| Driving Pressure (cmH2O) | 15.70 [11.33, 18.00] | 15.00 [11.00, 19.57] | 0.705 |
| Tidal volume (ml/kg PBW) | 5.29 [3.99, 7.23] | 5.44 [3.84, 7.24] | 0.882 |
| Respiratory rate (breaths/min) | 25.00 [20.04, 34.18] | 24.00 [19.40, 30.00] | 0.079 |
| Compliance (ml/cm H2O) | 23.04 [12.72, 33.25] | 20.55 [13.37, 29.74] | 0.066 |
| **Further rescue therapy, n (%)** |  |  |  |
| Inhaled nitric oxide | 74 (62.2) | 116 (75.3) | 0.027 |
| Prone positioning | 87 (73.1) | 117 (76.0) | 0.689 |
| **Organ failure at ECMO initiation, n (%)** |  |  |  |
| Coagulation | 19 (16.0) | 72 (46.8) | <0.001 |
| Liver | 7 (5.9) | 30 (19.5) | 0.002 |
| Cardiovascular | 119 (100.0) | 154 (100.0) | NA |
| CNS | 62 (52.1) | 78 (50.6) | 0.90 |
| Renal (KDIGO 3) | 0 (0.0) | 154 (100.0) | <0.001 |
| **Septic shock, n (%)** | 40 (33.6) | 112 (72.7) | <0.001 |
| **CFH (mg/dl)** | 6.00 [3.00, 9.00] | 9.35 [5.00, 16.00] | <0.001 |
| **Haptoglobin (g/l)** | 2.05 [1.14, 2.90] | 1.20 [0.48, 2.34] | <0.001 |
| **Serum creatinine level (mg/dl)** | 0.98 [0.73, 1.26] | 2.10 [1.48, 3.09] | <0.001 |
| **Renal replacement therapy (RRT) , n (%)** | 0 (0.0) | 149 (96.7) | <0.001 |
| **Mortality within 28 days after ECMO initiation, n (%)** | 27 (22.7) | 76 (49.4) | <0.001 |

Data are expressed as median [25%, 75% quartiles] or frequencies [%], as appropriate. *P-values were calculated using the exact Wilcoxon-Mann-Whitney test and the Fisher’s exact test, as appropriate. Abbreviations: SOFA = Sequential Organ Failure Assessment, SAPS = Simplified Acute Physiology Score, ECMO = Extracorporeal membrane oxygenation, PIP = Peak Inspiratory Pressure, Pplateau = Plateau pressure, PEEP = Positive End-Expiratory Pressure.

# Supplemental Figures

Figure S1: Distribution of CFH concentration of the study population. Intervals are set at 1mg/dl and are left-closed. Nine patients had undetectable CFH concentrations (3.3% [95% CI, 1.6-6.4]).

Figure S2: Distribution of haptoglobin concentration of the study population. Intervals are set at 0.1g/l and are left-closed.

Figure S3: Detailed overview of KDIGO 3 AKI components (A) and indications for RRT in patients with acute RRT at ECMO initiation (B). The proportion of patients with 95% CI are presented. *In the majority of patients with acute RRT, more than one criterion for acute RRT was present at ECMO initiation. For better visualization and interpretation, only the clinically most important criterion was presented. **Severe hyperkalemia or rapidly rising potassium levels.
